# Preoperative Platelet Count Correlates With Postoperative Perineural Invasion on Specimen in Patients Treated With Radical Prostatectomy

**DOI:** 10.3389/fonc.2022.906936

**Published:** 2022-06-07

**Authors:** Fangming Wang, Fei Liu, Jing Liang, Feiya Yang, Nianzeng Xing

**Affiliations:** ^1^ State Key Laboratory of Molecular Oncology, National Cancer Center/National Clinical Research Center for Cancer/Cancer Hospital, Chinese Academy of Medical Sciences and Peking Union Medical College, Beijing, China; ^2^ Department of Urology, National Cancer Center/National Clinical Research Center for Cancer/Cancer Hospital, Chinese Academy of Medical Sciences and Peking Union Medical College, Beijing, China; ^3^ Department of Pathology, National Cancer Center/National Clinical Research Center for Cancer/Cancer Hospital, Chinese Academy of Medical Sciences and Peking Union Medical College, Beijing, China; ^4^ Department of Urology, Shanxi Province Cancer Hospital/Shanxi Hospital Affiliated to Cancer Hospital, Chinese Academy of Medical Sciences/Cancer Hospital Affiliated to Shanxi Medical University, Taiyuan, China

**Keywords:** platelet, perineural invasion, radical prostatectomy, correlation, specimen

## Abstract

**Objective:**

It has been reported that perineural invasion (PNI) after radical prostatectomy (RP) is associated with unfavorable prostate cancer (PCa) prognosis. However, the clinicopathological factors especially hematological parameters that influenced PNI remain unknown. Our aim was to explore the relationship between clinicopathological parameters and PNI in patients who underwent RP.

**Methods:**

A total of 348 patients with PCa who underwent RP at our center between 2018 and 2021 were consecutively collected. We divided them into non-PNI and PNI groups based on PNI status and compared clinicopathological characteristics including hematological parameters between non-PNI and PNI groups. The association of clinicopathological parameters including whole blood parameters, age, body mass index (BMI), hypertension, diabetes mellitus, prostate-specific antigen (PSA), ISUP (International Society of Urological Pathology) grade, pathological stage T (pT), and neoadjuvant hormonal therapy (NHT) with PNI was determined by univariate and multivariate logistic regression analyses.

**Results:**

The pathological results of the RP specimen consisted of 254 (73.0%) patients with PNI and 94 (27.0%) cases without PNI. The level of PSA, percentages of advanced pT and grade, positive surgical margin rate, and vessel carcinoma embolus rate were significantly higher in the PNI group when compared with non-PNI counterpart (p = 0.007, p < 0.001, p < 0.001, p < 0.001, and p < 0.001, respectively). Among the whole blood parameters, only platelet count and plateletcrit were significantly different [216 (178.8–252.0) vs. 200.5 (173.5–236.5), p = 0.04; 0.0021 (0.0018–0.0025) vs. 0.0020 (0.0017–0.0023), p = 0.008, respectively]. Univariate logistic regression analysis demonstrated that platelet, ISUP, and pT were all positively correlated with the presence of PNI (T3 vs. T1, odds ratio (OR) = 2.029, p = 0.020; OR = 1.697, p < 0.001; OR = 3.836, p < 0.001). In the stepwise multivariate regression analysis, the association between platelet and PNI remained significant (T2 vs. T1, OR = 2.171, 95% CI: 1.082–4.354, p = 0.029; T3 vs. T1, OR = 2.595, 95% CI: 1.259–5.349, p = 0.010) after adjusting for confounding factors including age, BMI, hypertension, diabetes mellitus, PSA, ISUP, pT, and NHT.

**Conclusions:**

The study first revealed that platelet count rather than other whole blood parameters was independently associated with the presence of PNI in patients with PCa, suggesting that platelets might play an essential role in PCa aggressiveness.

## Introduction

Prostate cancer (PCa) is the second most common cancer and the fifth most common cause of cancer death among men worldwide in 2020 (1). Radical prostatectomy (RP) is appropriate for men with intermediate- and high-risk diseases in whom RP prevents further metastatic seeding of potentially lethal PCa cell clones. Small series have suggested that it might be appropriate to offer RP to men with low metastatic burden as part of a multimodal therapeutic approach (2). Postoperative histopathology results of RP including pathologic T stage (pT), Gleason score, and positive surgical margin (PSM) are of crucial importance to clarify the stage, make decisions for adjuvant treatment, and predict biochemical recurrence and prognosis after RP (3, 4). More and more pathological information was explored and added to the current models for improving the predictive power of prognosis and the precise timing of adjuvant treatment. Perineural invasion (PNI), a routine evaluation in RP specimens (a mean frequency of 62.2% and up to 80%), has recently attracted more and more attention recently (3). PNI is defined as cancer tracking along or around a nerve within the perineural space; it can be observed in the absence of lymphatic or vascular invasion (5). There was no doubt that the presence of PNI in the RP specimen was correlated with multiple adverse clinicopathological factors (6–13). Although controversial (6–9), there were abundant evidence supporting that PNI could be used as an independent predictor of biochemical recurrence in patients with PCa who underwent RP (10–13).

The exact mechanism of PNI is still unclear although PNI is usually seen in cases of PCa; the factors associated with the ability of PCa cells to permeate nerves are not completely elucidated. Some studies focused on the perineural space and the interaction between PCa cells and nerves, suggesting that the perineural space may be a tumor microenvironment (TME) that promotes both cancer spread and growth (5, 14). As one of the important stromal components in the TME, platelets have attracted attention in recent years, and their interaction with cancer cells could promote the spread of PCa cancer cells with less resistance (15, 16). However, it remains largely unknown whether platelet is correlated with PNI in PCa. Until now, there was no study that systematically and comprehensively investigated the correlation between platelet and other whole blood parameters with PNI and other clinicopathological features of PCa.

Hence, the aim of the current study was to identify the association of comprehensive whole blood parameters with the presence of PNI of RP specimen in the Chinese patients with PCa.

## Materials and Methods

### Collection of Patients’ Clinicopathological Data

A total of 348 patients with PCa who had been pathologically diagnosed as PCa by biopsy and underwent laparoscopic RP and pelvic lymph node dissection at our institution between May 2018 and June 2021 were reviewed. Exclusion criteria of the study were the presence of infection, hematologic diseases, severe hepatic and/or renal insufficiency, hypersplenism, hyperthyroidism, cardiovascular disease, medical history of other malignancies, and receiving any radiotherapy or chemotherapy.

All clinicopathological data including age, body mass index (BMI), hypertension, diabetes mellitus, prostate-specific antigen (PSA), neoadjuvant hormonal therapy (NHT), operative time, blood loss, preoperative whole blood parameters, and postoperative pathological results including ISUP (International Society of Urological Pathology) grade and pathological stage T (pT) were collected from medical records. All RP samples were routinely sent to the pathological department for diagnosis. The cancer grade assessment was performed according to the ISUP 2014 classification system (17). The pT was evaluated on the basis of a prostatectomy specimen according to the American Joint Committee on Cancer (AJCC) TNM classification of malignant tumors in 2017 (AJCC, pT2–T4, Nx,0,1) (18). The pathology of the presence of PNI, PSM, and vessel carcinoma embolus (VCE) was reviewed by two pathologists. For controversial cases, a third pathologist was invited to reach group agreement.

### Hematological Parameter Measurement

Morning venous blood samples were taken on admission at the nurse station after 8-h fasting and before biopsy without any treatment. The serum PSA value and the whole blood parameters were measured; the latter included white blood cell (WBC), neutrophils (%), neutrophil counts, lymphcytes (%), lymphocyte counts, monocytes (%), monocyte counts, eosinophils (%), eosinophil counts, basophils (%), basophil counts, red blood cell (RBC), hemoglobin (Hb), hematocrit (Hct), mean corpuscular volume (MCV), mean corpuscular hemoglobin (MCH), mean corpuscular hemoglobin concentration (MCHC), red blood cell volume distribution width-standard deviation (RDW-SD), red blood cell volume distribution width–coefficient of variability (RDW-CV), platelet, mean platelet volume (MPV), platelet–large cell ratio (P-LCR), platelet distribution width (PDW), and plateletcrit.

### Statistical Analysis

Data were expressed as number (percentage) for categorical variables and means ± SD or median with interquartile range for continuous variables. Categorical variables were analyzed by χ2-test. The differences between continuous variables were analyzed by unpaired t-tests, Mann–Whitney U-tests, or one way ANOVA as appropriate. All PCa subjects were divided into two groups according to PNI status: non-PNI and PNI groups, and clinicopathological characteristics especially all hematological parameters were compared between the two groups. The platelet was stratified into tertiles as follows: the first tertile group (T1, <192 × 10^9^/L (33.3th percentile), *n* = 118); the second tertile group [T2, 192–236 × 10^9^/L (33.3–66.7th percentile), *n* = 116]; and the third tertile group (T3, >237 × 10^9^/L [66.7–100th percentile); *n* = 114], and clinicopathological characteristics were analyzed in the different tertiles of platelet. The binary logistic regression model (univariate and multivariate analysis) was used to evaluate the association between clinicopathological parameters including platelet, age, BMI, hypertension, diabetes mellitus, PSA, ISUP grade, pT, and NHT with PNI. A p-value <0.05 (two-sided) was considered significant for all tests. The statistical analyses were performed with SPSS version 22.0 software (SPSS Inc., Chicago, IL, USA).

## Results

### Characteristics of PNI and Non-PNI Patients

**Figure 1:**
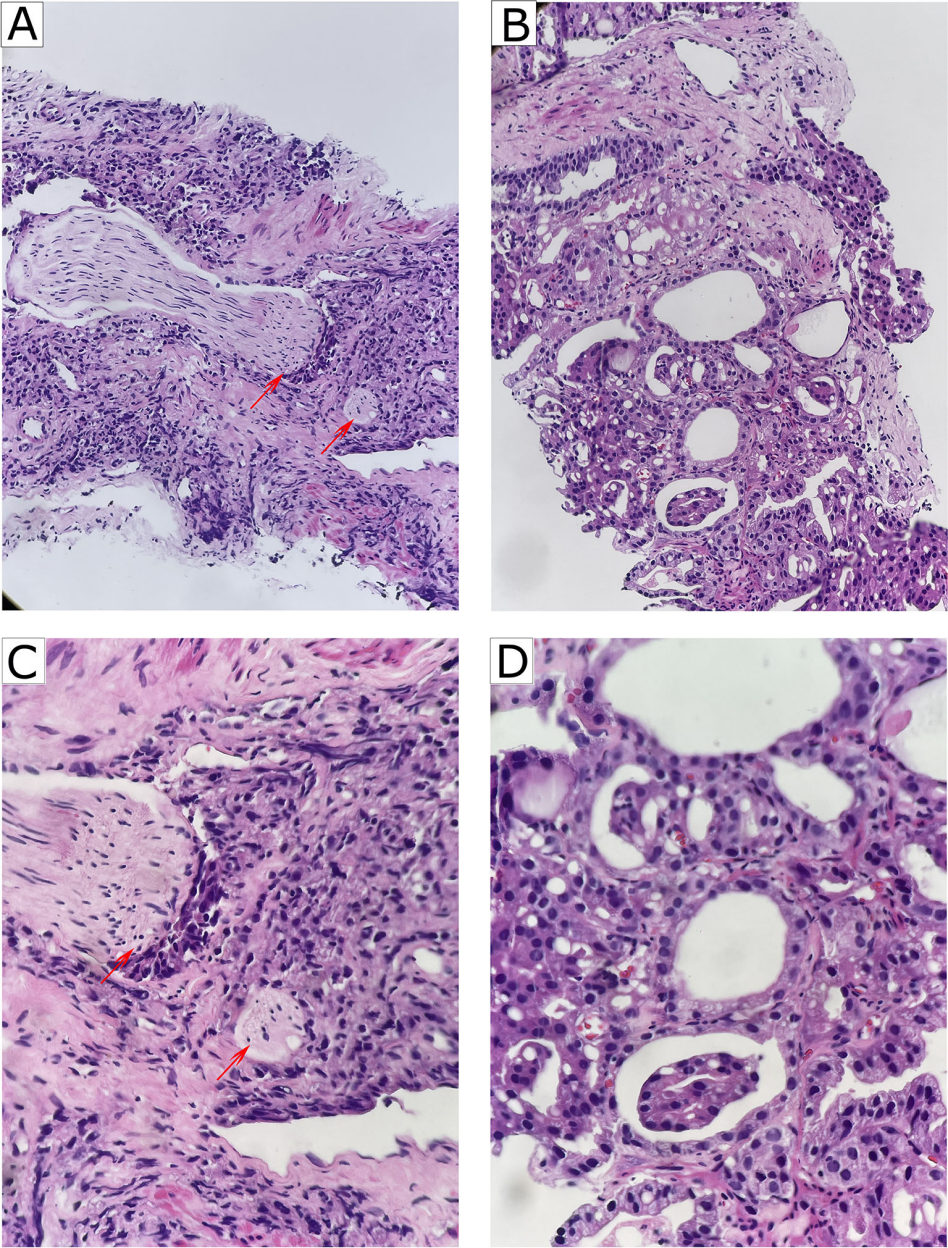
Representative of PNI and non-PNI sections of HE staining from patients with PCa. **(A)** Representative image of PNI (HE, ×200). The nerves (red arrow heads) were invaded by surrounding cancer cells. **(B)** Representative image of non-PNI (HE, ×200) for comparison. **(C)** Higher magnification of A (HE, ×400) showing that the nerves (red arrow heads) were invaded by surrounding cancer cells. **(D)** Higher magnification of **(B)** (HE, ×400). PNI, perineural invasion; PCa, prostate cancer; HE, hematoxylin-eosin.

**Figure 2:**
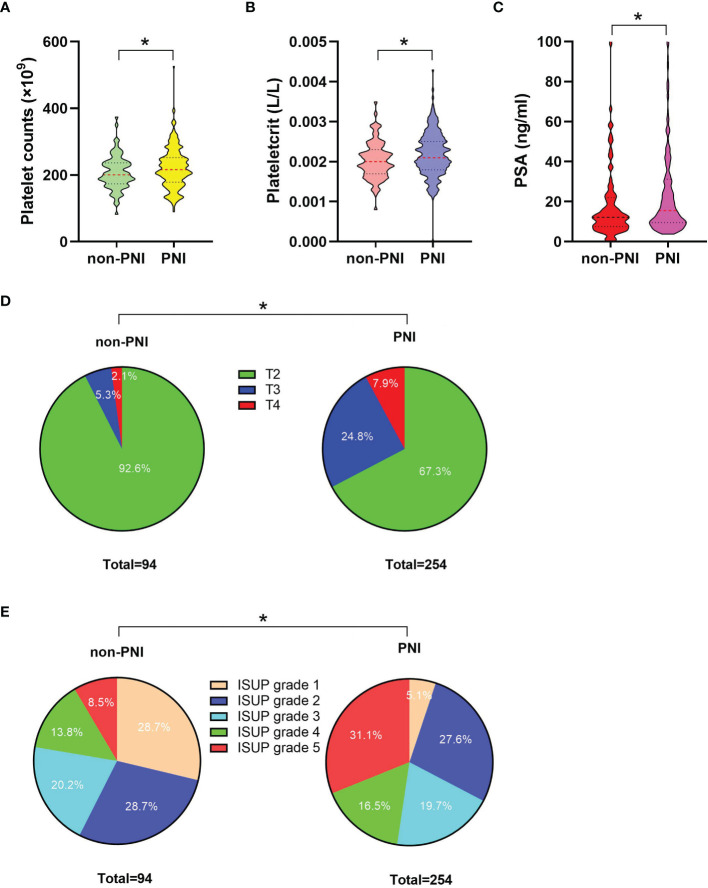
The comparisons of platelet count, plateletcrit, PSA level, the percentage distributions of pT, and IUSP grade between the non-PNI (n = 94) and PNI (n = 254) groups. **(A)** Comparison of platelet count between the non-PNI and PNI groups; median (interquartile range) was measured and showed in a violin plot (the dotted line); statistical significance was determined by the Mann–Whitney U-test. **(B)** Comparison of plateletcrit between the non-PNI and PNI groups, median (interquartile range) was measured and showed in a violin plot (the dotted line), statistical significance was determined by the Mann–Whitney U-test. **(C)** Comparison of PSA levels between the non-PNI and PNI groups; median (interquartile range) was measured and showed in a violin plot (the dotted line); statistical significance was determined by the Mann–Whitney U-test. **(D)** Comparison of pT distribution (including T2, T3, and T4 percentages) between the non-PNI and PNI groups; a pie plot was generated to visualize the proportional change of the numerical data; statistical significance was determined by χ2-test; **(E)** Comparison of ISUP grade distribution (including proportions of ISUP grades 1, 2, 3, 4, and 5) between the non-PNI and PNI groups; a pie plot was generated to visualize the proportional change of the numerical data; statistical significance was determined by χ2-test. Values are expressed as median with interquartile range or number (percentage); *, P < 0.05. PSA, prostate-specific antigen; pT, pathological stage T; ISUP, International Society of Urological Pathology; PNI, perineural invasion.

The clinicopathological characteristics and whole blood parameters of the enrolled subjects were shown in **Table 1**. Of the total 348 eligible RP cases, the mean values of the clinical factors were 66.2 ± 6.6 years for age and 25.1 ± 3.2 for BMI; the median PSA was 14.0 (8.7–29.6); 135 cases (38.8%) received NHT therapy; 258 (74.1%) were pT2, 68 (19.5%) were pT3, and 22 (6.3%) were pT4; lymph node involvement occurred in 24 cases (6.9%), and metastasis occurred in 19 cases (5.5%). The percentages of ISUP grades from 1 to 5 were 40 (11.5%), 97 (27.9%), 69 (19.8%), 55 (15.8%), and 87 (25%). The PSM and VCE rate were 94 (27%) and 54 (15.5%), respectively. It is noteworthy that PNI was presented in 254 cases (73.0%) and absent in 94 cases (27%). The representative hematoxylin-eosin (HE) staining images of PNI and non-PNI were shown in **Figures 1A–D**. No significant differences in age, BMI, hypertension, diabetes, NHT percentage, operative time, blood loss, or complete blood count except platelet were observed between the non-PNI and PNI groups. Platelet count and plateletcrit were significantly higher in the PNI group than that in the non-PNI group [216 (178.8–252.0) vs. 200.5 (173.5–236.5), p = 0.04; 0.0021 (0.0018–0.0025) vs. 0.0020 (0.0017–0.0023), p = 0.008, respectively] (**Figures 2A, B**). The level of PSA was significantly higher in the PNI group compared to the non-PNI counterpart [15.5 (9.5–31.5) vs. 12.2 (7.6–22.0), p = 0.007] (**Figure 2C**). In addition, as shown in **Figures 2D, E**, the percentages of advanced pT (T3 + T4) and high IUSP grades (4 + 5) were significantly higher in the PNI group when compared with non-PNI counterpart (32.7% vs. 7.4%, p < 0.001; 47.6% vs. 22.3%, p < 0.001). Furthermore, there were also significant differences in the PSM and VCE rates between the two groups [84 (33.1%) vs. 10 (10.6%), p < 0.001; 51 (20.1%) vs. 3 (3.2%), p < 0.001].

**Table 1:** Baseline characteristics of the PCa subjects underwent RP according to the PNI stratification.

	All subjects(*n* = 348)	PNI (n = 254)	Non-PNI (n = 94)	P-value
**Demographic characteristics**				
Age (years)	66.2 ± 6.6	66.1 ± 6.5	66.4 ± 6.6	0.757
BMI (kg/m^2^)	25.1 ± 3.2	25.1 ± 3.1	25.1 ± 3.3	0.956
Hypertension [*n* (%)]	154 (44.3)	106 (41.7)	48 (51.1)	0.113
Diabetes mellitus [*n* (%)]	69 (19.8)	51 (20.1)	18 (19.1)	0.859
**Clinicopathological characteristics**				
PSA (ng/ml)	14.0 (8.7–29.6)	15.5 (9.5–31.5)	12.2 (7.6–22.0)	**0.007**
NHT	135 (38.8)	97 (38.2)	38 (40.4)	0.633
Pathological Stage				**<0.001**
T2	258 (74.1)	171 (67.3)	87 (92.6)	
T3	68 (19.5)	63 (24.8)	5 (5.3)	
T4	22 (6.3)	20 (7.9)	2 (2.1)	
N	24 (6.9)	23 (9.1)	1 (1.1)	**0.009**
M	19 (5.5)	16 (6.3)	3 (3.2)	0.260
ISUP grade [n (%)]				**<0.001**
1	40 (11.5)	13 (5.1)	27 (28.7)	
2	97 (27.9)	70 (27.6)	27 (28.7)	
3	69 (19.8)	50 (19.7)	19 (20.2)	
4	55 (15.8)	42 (16.5)	13 (13.8)	
5	87 (25)	79 (31.1)	8 (8.5)	
PSM [n (%)]	94 (27.0)	84 (33.1)	10 (10.6)	**<0.001**
VCE [n (%)]	54 (15.5)	51 (20.1)	3 (3.2)	**<0.001**
Operative time (min)	165.1 ± 58.2	165.6 ± 60.2	163.5 ± 52.8	0.762
Evaluated blood loss (ml)	59.5 ± 94.2	62.8 ± 98.4	50.7 ± 81.7	0.288
Transfusion [n (%)]	6 (1.7%)	5 (2.0)	1 (1.1)	0.567
**Whole blood parameters**				
WBC (×10^9^/L)	6.2 ± 1.5	6.2 ± 1.6	6.1 ± 1.5	0.436
Neutrophils (%)	61.4 ± 9.3	61.5 ± 9.2	60.9 ± 9.7	0.617
Neutrophil counts (×10^9^/L)	3.8 ± 1.3	3.9 ± 1.3	3.8 ± 1.3	0.459
Lymphocytes (%)	29.7 ± 8.6	29.7 ± 8.4	29.9 ± 9.1	0.885
Lymphocyte counts (×10^9^/L)	1.8 ± 0.6	1.8 ± 0.6	1.8 ± 0.6	0.546
Monocytes (%)	6.1 ± 1.5	6.1 ± 1.5	6.3 ± 1.6	0.202
Monocyte counts (×10^9^/L)	0.4 ± 0.1	0.4 ± 0.1	0.4 ± 0.1	0.856
Eosinophils (%)	2.2 ± 1.9	2.1 ± 1.8	2.3 ± 2.1	0.455
Eosinophil counts (×10^9^/L)	0.1 ± 0.1	0.1 ± 0.1	0.1 ± 0.1	0.757
Basophils (%)	0.6 ± 0.3	0.6 ± 0.3	0.6 ± 0.3	0.823
Basophil counts (×10^9^/L)	0.04 ± 0.02	0.04 ± 0.02	0.04 ± 0.02	0.669
RBC (×10^12^/L)	4.8 ± 0.5	4.8 ± 0.5	4.7 ± 0.5	0.308
Hb (g/L)	147.3 ± 13.8	147.8 ± 13.7	146.1 ± 14.3	0.323
Hct (L/L)	0.43 ± 0.04	0.44 ± 0.04	0.43 ± 0.04	0.159
MCV (fl)	91.6 ± 5.0	91.7 ± 5.2	91.3 ± 4.4	0.522
MCH (pg)	31.3 ± 3.5	31.1 ± 1.9	31.7 ± 5.9	0.117
MCHC (g/L)	338.8 ± 19.4	338.1 ± 21.7	340.8 ± 10.4	0.247
RDW-SD (fl)	42.0 ± 3.0	42.0 ± 3.0	41.8 ± 3.1	0.460
RDW-CV (%)	12.6 ± 0.8	12.6 ± 0.9	12.5 ± 0.8	0.716
PLT (×10^9^/L)	211.5 (177.3–248.0)	216 (178.8–252.0)	200.5 (173.5–236.5)	**0.04**
MPV (fl)	10.0 ± 1.1	10.0 ± 1.1	9.9 ± 0.9	0.479
P-LCR (%)	24.7 ± 7.4	25.0 ± 7.5	24.1 ± 7.3	0.345
PDW (fl)	11.3 ± 2.1	11.4 ± 2.2	11.1 ± 2.0	0.268
PCT (L/L)	0.0021 (0.0018–0.0025)	0.0021 (0.0018–0.0025)	0.0020 (0.0017–0.0023)	**0.008**

Data are expressed as n (%), mean ± SD, or median (interquartile range). The bold value indicated statistical significance. PCa, prostate cancer; RP, radical prostatectomy; PNI, perineural invasion; BMI, body mass index; PSA, prostate-specific antigen; NHT, neoadjuvant hormonal therapy; ISUP, International Society of Urological Pathology; PSM, positive surgical margin; VCE, vessel carcinoma embolus; WBC, white blood cell; RBC, red blood cell; Hb, hemoglobin; Hct, hematocrit; MCV, mean corpuscular volume; MCH, mean corpuscular hemoglobin; MCHC, mean corpuscular hemoglobin concentration; RDW-SD, red blood cell volume distribution width–standard deviation; RDW-CV, red blood cell volume distribution width–coefficient of variability; PLT, platelet; MPV, mean platelet volume; P-LCR, platelet–large cell ratio; PDW, platelet distribution width; PCT, plateletcrit.

### Relation of Platelet Count With the Clinicopathological Characteristics of PCa

Patients with PCa who underwent RP were stratified into three groups according to the platelet count level tertiles as mentioned due to the evident difference in platelets between the PNI and non-PNI groups. Statistically significant differences in age, PNI, and platelet-related parameters including MPV, P-LCR, PDW, and PCT were found among the three different groups (p < 0.001, p = 0.030, p < 0.001, p < 0.001, p < 0.001, and p < 0.001, respectively) (**Table 2**). Specifically, PNI percentages increased with the elevation of the platelet count level, from 65.3%, 73.3%, up to 80.7%. However, we did not observe any significant difference regarding the PSA, pT, lymph node involvement, metastasis, ISUP grade, or VCE among the three platelet groups (p = 0.295, p = 0.065, p = 0.200, p = 0.687, p = 0.424, and p = 0.868, respectively).

**Table 2:** Clinical characteristics of PCa subjects according to tertiles of platelet levels.

		Platelet Tertiles		P-value
	T1 (<192 × 10^9^/L, n = 118)	T2 192–236 × 10^9^/L, n = 116)	T3 (>237 × 10^9^/L, n = 114)	
Age (years)	68.1 ± 5.8	65.8 ± 6.5	64.6 ± 6.8	**<0.001**
PSA (ng/ml)	26.7 ± 37.1	23.6 ± 31.8	31.8 ± 47.1	0.295
Pathological Stage				0.065
T2	91 (77.1)	86 (74.1)	81 (71.1)	
T3	18 (15.3)	28 (24.1)	22 (19.3)	
T4	9 (7.6)	2 (1.7)	11 (9.6)	
N				0.200
N0	106 (89.8)	109 (94.0)	109 (95.6)	
N1	12 (10.2)	7 (6.0)	5 (4.4)	
M				0.687
M0	112 (94.9)	108 (93.1)	109 (95.6)	
M1	6 (5.1)	8 (6.9)	5 (4.4)	
ISUP grade [n (%)]				0.424
1	9 (7.6)	16 (13.8)	15 (13.2)	
2	32 (27.1)	35 (30.2)	30 (26.3)	
3	22 (18.6)	26 (22.4)	21 (18.4)	
4	18 (15.3)	19 (16.4)	18 (15.8)	
5	37 (31.4)	20 (17.2)	30 (26.3)	
PNI [n (%)]	77 (65.3)	85 (73.3)	92 (80.7)	**0.030**
VCE	19 (16.1)	16 (13.8)	18 (15.8)	0.868
MPV (fl)	10.3 ± 1.4	9.9 ± 0.7	9.7 ± 0.7	**<0.001**
P-LCR (%)	27.8 ± 8.9	23.8 ± 6.0	22.5 ± 5.9	**<0.001**
PDW (fl)	12.1 ± 2.8	11.0 ± 1.5	10.7 ± 1.4	**<0.001**
PCT (L/L)	0.0016 ± 0.0003	0.0021 ± 0.0002	0.0027 ± 0.0004	**<0.001**

Data are expressed as n (%), mean ± SD, or median (interquartile range). The bold value indicated statistical significance. PCa, prostate cancer; PSA, prostate-specific antigen; ISUP, International Society of Urological Pathology; PNI, perineural invasion; VCE, vessel carcinoma embolus; MPV, mean platelet volume; P-LCR, platelet–large cell ratio; PDW, platelet distribution width; PCT, plateletcrit.

### Correlation of Platelet Count and Other Clinicopathological Variables With the Presence of PNI

To evaluate the correlation of platelet count and other clinical variables with PNI, in the present study, a univariate and multivariate logistic regression analysis was performed. As shown in **Table 3**, parameters including platelet, ISUP, and pT were found to be significantly and positively correlated with the presence of PNI (T3 vs. T1, OR = 2.029, 95% CI: 1.119–3.680, p = 0.020; OR = 1.697, 95% CI: 1.396–2.063, p < 0.001; OR = 3.836, 95% CI: 2.504–5.876, p < 0.001) in univariate logistic regression analyses. In the stepwise multivariate regression analysis (**Table 4**), we gradually added and adjusted confounding factors from model 1 to model 4 and ultimately revealed that the correlations of platelet, ISUP, and pT with PNI remained significant (T2 vs. T1, OR = 2.171, p = 0.029; T3 vs. T1, OR = 2.595, p = 0.010; OR = 1.509, p = 0.001; OR = 3.220, p < 0.001) after adjustment for confounding factors. In particular, the positive correlation between platelets and PNI became increasingly evident when confounders were gradually adjusted. Furthermore, the multivariate regression analysis showed that NHT was negatively and independently correlated with the presence of PNI (OR = 0.420, 95% CI: 0.220–0.804, p = 0.009). However, no significant correlation between PSA and PNI was observed (OR = 1.002, 95% CI: 0.992–1.013, p = 0.704).

**Table 3:** Univariate analysis for PCa patients with PNI.

	Univariate Mode		P-value
	OR	95% CI	
PLT (×10^9^/L)			
T1(<)	reference		
T2(−)	1.406	0.803–2.463	0.234
T3(>)	2.029	1.119–3.680	**0.020**
Age	0.994	0.959–1.031	0.756
BMI	0.998	0.926–1.075	0.956
Hypertension	0.682	0.424–1.096	0.114
Diabetes mellitus	1.056	0.580–1.920	0.859
PSA	1.009	1.000–1.019	0.059
ISUP	1.697	1.396–2.063	**0.000**
T stage	3.836	2.504–5.876	**0.000**
NHT	0.889	0.547–1.443	0.633

Univariate regression analyses are applied. The bold value indicated statistical significance. PCa, prostate cancer; PNI, perineural invasion; PLT, platelet; BMI, body mass index; PSA, prostate-specific antigen; ISUP, International Society of Urological Pathology; NHT, neoadjuvant hormonal therapy.

**Table 4:** Multivariate analysis to identify the independent correlation between PLT and PNI of PCa.

Models	Variables		Multivariate Mode		P-value
			OR	95% CI	
1		unadjusted			
	PLT (×10^9^/L)	T1(<)	Reference		
		T2(−)	1.406	0.803–2.463	0.234
		T3(>)	2.029	1.119–3.680	**0.020**
2		Model 1+covariates			
	PLT (×10^9^/L)	T1(<)	Reference		
		T2(−)	1.463	0.823–2.601	0.195
		T3(>)	2.342	1.249–4.390	**0.008**
3		Model 2 + PSA + ISUP + T stage			
	PLT (×10^9^/L)	T1(<)	Reference		
		T2(−)	2.076	1.054–4.090	**0.035**
		T3(>)	2.547	1.255–5.169	**0.010**
4		Model 3+NHT			
	PLT (×10^9^/L)	T1(<)	Reference		
		T2(−)	2.171	1.082–4.354	**0.029**
		T3(>)	2.595	1.259–5.349	**0.010**
	Age		1.017	0.973–1.063	0.460
	BMI		0.961	0.875–1.056	0.406
	Hypertension		0.566	0.317–1.013	0.055
	Diabetes mellitus		1.250	0.607–2.573	0.545
	PSA		1.002	0.992–1.013	0.704
	ISUP		1.509	1.188–1.917	**0.001**
	T stage		3.220	2.041–5.081	**0.000**
	NHT		0.420	0.220–0.804	**0.009**

Multivariate regression stepwise models are shown. The bold value indicated statistical significance. The dependent variable was PNI of PCa. Model 1 was unadjusted. Model 2 was corrected for covariates including age, BMI, hypertension, and diabetes mellitus. Model 3 was additionally corrected for PSA, ISUP, stage T based on Model 2; Model 4 was additionally corrected for NHT based on Model 3. PLT, platelet; PNI, perineural invasion; PCa, prostate cancer; PSA, prostate-specific antigen; ISUP, International Society of Urological Pathology; NHT, neoadjuvant hormonal therapy; BMI, body mass index; PSA, prostate-specific antigen.

## Discussion

Our study, for the first time, explored the correlation between clinicopathological parameters and PNI in patients who underwent RP and demonstrated that platelet count was independently and positively associated with the presence of PNI among all whole blood parameters. Therefore, the current research may have clinical implications for the assessment of PCa aggressiveness from the perspective of routine blood tests. Moreover, our study provided novel clues to explore the mechanisms of the roles the platelet played in PCa cell invasion.

PNI, a pathologic feature defined as the invasion of cancer cells in, around, and through the nerves, is an indicator of poor prognosis in PCa (19). Different from lymphatic or vascular involvement (5), PNI occurs within all three layers of the nerve sheath (epineurium, perineurium, and endoneurium), which means that cancer cells could track along nerves beyond the predicted anatomic boundaries of primary tumors, often resulting in residual tumor within the body after surgery. PNI has been reported to be correlated with adverse pathological features, elevated biochemical recurrence rates, increased risk of bone metastasis, and poor overall survival in patients with PCa (20–22). PNI is highly prevalent in PCa; one study reported that PNI was observed in up to 75% of surgical resection specimens (23). In our study, PNI was presented in 73.0% of the prostatectomy specimens, which was consisted with the mentioned data. Our data showed that patients with PNI had higher PSA levels, more advanced stage and grade, higher PSM rate and VCE rate, compared to their counterparts without PNI. The explanation for this phenomenon was that PNI is an ominous clinical and pathological characteristic of PCa, which has been associated with adverse pathological features (19).

In our study, among all whole blood parameters, only platelet count and plateletcrit in the PNI group were significantly higher than those in non-PNI group. Chronic inflammation promotes cancer progression and metastasis (24). Changes in peripheral blood counts can partly reflect inflammatory responses in cancer patients. Some studies suggested that platelets can mediate cancer cell growth, dissemination, and angiogenesis (25–29). Platelet–tumor cell interactions could prime tumor cells for subsequent extravasation and metastasis through platelet-derived transforming growth factor-β (TGFβ)-mediated pathways in cancer cells (26, 27). In addition, aggregation of platelets around tumor cells could protect tumor cells from immune surveillance, which, in part, enhances cancer cell invasion (28, 29). Moreover, platelets can change the phenotype of epithelial tumors, which is known as the epithelial–mesenchymal transition, conferring stem cell properties to tumor cells associated with increased mobility (30). Regarding studies of the relationship between platelets and PCa aggressiveness, recently, Rudzinski et al. (31) reported that platelets could enhance the invasion of androgen receptor-negative PCa cells; Brady et al. identified that platelet-coated circulating tumor cells existed in 29.5% of patients with metastatic PCa (32), and Chai et al. claimed that platelet-coated circulating tumor cells could predict the worst prognosis of a subset of patients with metastatic castration-resistant PCa (33).

Our study first showed that the percentage of PNI gradually increased (from 65.3%, 73.3%, to 80.7%) with increasing platelet count (from T1, T2, to T3), indicating that platelets play crucial roles in helping tumor cells pass through neural metastasis when they interact with each other. The D’Amico risk stratification system classifies patients with PCa into low-, intermediate-, and high-risk groups based on PSA level, clinical tumor stage, and Gleason score at diagnosis (34). Among the three parameters, our study demonstrated that ISUP grade (converted from the Gleason score) and pT (more accurate than clinical stage) were found to be significantly and positively correlated with the presence of PNI. In addition, our results showed that NHT was negatively and independently correlated with the presence of PNI, suggesting that NHT could result in less frequent invasion of the perineural spaces, which consisted with one previous study (35). It should be emphasized that platelet was the only whole blood parameter that was independently and positively correlated with the presence of PCa PNI. The present study might have two potential clinical hints. First, the blood platelet count could be used to predict the PNI of postoperative specimen and to estimate the prognosis; second, as platelets play crucial roles in PCa invasion and metastasis, maybe anti-platelet agent(s) could be recommended to patients with PCa with high platelet count to reduce the risk of PNI and metastasis.

## Conclusions

In conclusion, this study first demonstrated the clinicopathological characteristics that included whole blood parameters between PNI and non-PNI of patients with PCa who underwent RP and finally found that platelet count was positively and independently associated with the presence of PNI. There were two main limitations in our study. First, there might be selection bias because our data were derived from a single center, and studies from different institutions are needed for validation of these results. Second, the exact mechanism of platelet induced PNI was not explored in the current analysis.

## Data Availability Statement

The raw data supporting the conclusions of this article will be made available by the authors, without undue reservation.

## Ethics Statement

The studies involving human participants were reviewed and approved by Ethical Committee of National Cancer Center. Written informed consent for participation was not required for this study in accordance with the national legislation and the institutional requirements.

## Authors Contributions

Conceptualization: FW and NX; Formal analysis: FW and FL; Investigation: FW, FL, JL, and FY; Methodology: FW, FL, JL, and FY; Pathology analysis: JL; Supervision: NX; Writing—original draft: FW and FL; Writing—revision and editing: FW, FL, JL, FY, and XN. All authors contributed to the article and approved the submitted version.

## Funding

This work was supported by grants from National Natural Science Foundation of China (no. 81972400, 81772700), and Chinese Academy Medical Sciences Innovation Fund for Medical Sciences (no. 2019-I2M-1-003).

## Conflict of Interest

The authors declare that the research was conducted in the absence of any commercial or financial relationships that could be construed as a potential conflict of interest.

## Publisher’s Note

All claims expressed in this article are solely those of the authors and do not necessarily represent those of their affiliated organizations, or those of the publisher, the editors and the reviewers. Any product that may be evaluated in this article, or claim that may be made by its manufacturer, is not guaranteed or endorsed by the publisher.

## Abbreviations

PNI, perineural invasion; RP, radical prostatectomy; PCa, prostate cancer; BMI, body mass index; PSA, prostate-specific antigen; ISUP, International Society of Urological Pathology; NHT, neoadjuvant hormonal therapy; PSM, positive surgical margin; TME, microenvironment; AJCC, American joint committee on cancer; VCE, vessel carcinoma embolus; WBC, white blood cell; RBC, red blood cell; Hb, hemoglobin; Hct, hematocrit; MCV, mean corpuscular volume; MCH, mean corpuscular hemoglobin; MCHC, mean corpuscular hemoglobin concentration; RDW-SD, red blood cell volume distribution width–standard deviation; RDW-CV, red blood cell volume distribution width–coefficient of variability; MPV, mean platelet volume; P-LCR, platelet-large cell ratio; PDW, platelet distribution width.
